# Fatal Hemorrhagic Shock From Rupture of a Giant Uterine Artery Pseudoaneurysm 20 Years After Myomectomy: A Case Successfully Managed With Transcatheter Arterial Embolization

**DOI:** 10.7759/cureus.87814

**Published:** 2025-07-13

**Authors:** Toshiro Imamoto, Makoto Sawano

**Affiliations:** 1 Department of Emergency Medicine and Critical Care, Saitama Medical Center, Saitama Medical University, Kawagoe, JPN

**Keywords:** hemorrhagic shock, late complication, myomectomy, transcatheter arterial embolization, uterine artery pseudoaneurysm

## Abstract

A uterine artery pseudoaneurysm (UAP) is a rare and serious complication after gynecological surgery, and its rupture can cause fatal bleeding. In this report, a rare case of UAP rupture 20 years after myomectomy is presented, along with a discussion of the diagnosis and treatment strategy. A 54-year-old woman had undergone open hysterectomy for cervical myoma 20 years earlier. She was brought to the emergency room with sudden lumbar back pain and loss of consciousness and was diagnosed with a ruptured pseudoaneurysm of the right uterine artery by contrast-enhanced computed tomography and angiography. Transcatheter arterial embolization was performed, and her vitals were stabilized. Subsequent magnetic resonance imaging showed a pseudoaneurysm after a contained rupture that had become organic, and a portion of the nonorganized component remained. Six months later, a partial resection was performed by laparotomy. This case indicates the need for long-term postoperative follow-up and is reported here for comparison with previous reports.

## Introduction

Uterine artery pseudoaneurysms (UAPs) are rare following repeat curettage, abortion, cesarean section, uncomplicated vaginal delivery, or infection of the reproductive tract [1]. Once ruptured, they are dangerous and can lead to severe hemorrhage. Among these, pelvic surgery-related UAPs are usually unnoticed in the early postoperative period, as the interval between surgery and symptom onset is typically one week to three months [2,3]. Endovascular treatment has been increasingly selected in recent years due to its low invasiveness and fertility preservation, and it has a high success rate [4].

A case of spontaneous rupture of a uterine artery pseudoaneurysm 20 years after myomectomy, which was potentially fatal, is reported. Transcatheter arterial embolization (TAE) was performed for initial hemostasis, and abdominal hematoma removal and partial pseudoaneurysmectomy were performed six months later.

UAP rupture after myomectomy is rare [5]. The size and location of the aneurysm and the treatment strategy are reported, along with a discussion of the literature.

## Case presentation

The patient was a 54-year-old woman who, 20 years earlier, had visited a nearby gynecologist with complaints of back pain and lower abdominal distention. On close examination, she was found to have a giant cervical myoma with a maximum diameter of 20 cm. One month after diagnosis, an open hysterectomy was performed. The removed uterus weighed 4050 grams and had grown to the point that it was stuck in the pelvis. It was determined that uterine preservation was not possible, and surgery was performed to partially enucleate the myoma so that a small amount of myometrium remained on the pelvic side of the uterus. There were no abnormalities in the bilateral ovaries, and the ovaries and fallopian tubes were preserved. One week postoperatively, when the patient was defecating in the lavatory in the hospital room, a blood clot-like substance came out in a stalk-like shape. When it tore off, persistent and profuse genital bleeding was observed. The gynecologist identified arterial bleeding from the left vaginal segment and performed transvaginal suture hemostasis. At the same time, a retroperitoneal hematoma with a maximum diameter of 8 cm was also observed. The hematoma was monitored and had shrunk to 2 cm in size one month after surgery, at which point the patient was discharged from the hospital. The hematoma was almost completely absorbed at two months postoperatively, confirmed during an outpatient clinic visit. One year after surgery, plain computed tomography (CT) showed a 3 cm cyst in the right pelvis. It was difficult to determine whether it was an ovarian cyst or a pseudocyst caused by the remnant hematoma, but it was followed up as a right ovarian cyst on ultrasound examination. After that, the patient experienced hardening of the lower abdomen. She developed subjective symptoms of lower abdominal hardness, but as the symptoms did not worsen, she did not visit a hospital. In addition, constipation symptoms continued as they had before the surgery.

The night before her current admission to the hospital, she developed sudden lumbar pain, which resolved spontaneously. Therefore, on the day of admission, she went camping with her husband. While setting up a tent at the campsite, she collapsed. The patient was foaming at the mouth and unconscious and was transported by ambulance to her previous hospital. During transport, the patient’s consciousness improved, but she was disturbed and complained of back pain.

On arrival at the previous hospital, the patient’s vital signs were as follows: blood pressure could not be measured, the common carotid pulse was palpable, pulse rate was 130 beats per minute, respiratory rate was 24 breaths per minute, and SpO₂ was 99% (on 10 L/min oxygen).

**Figure 1:**
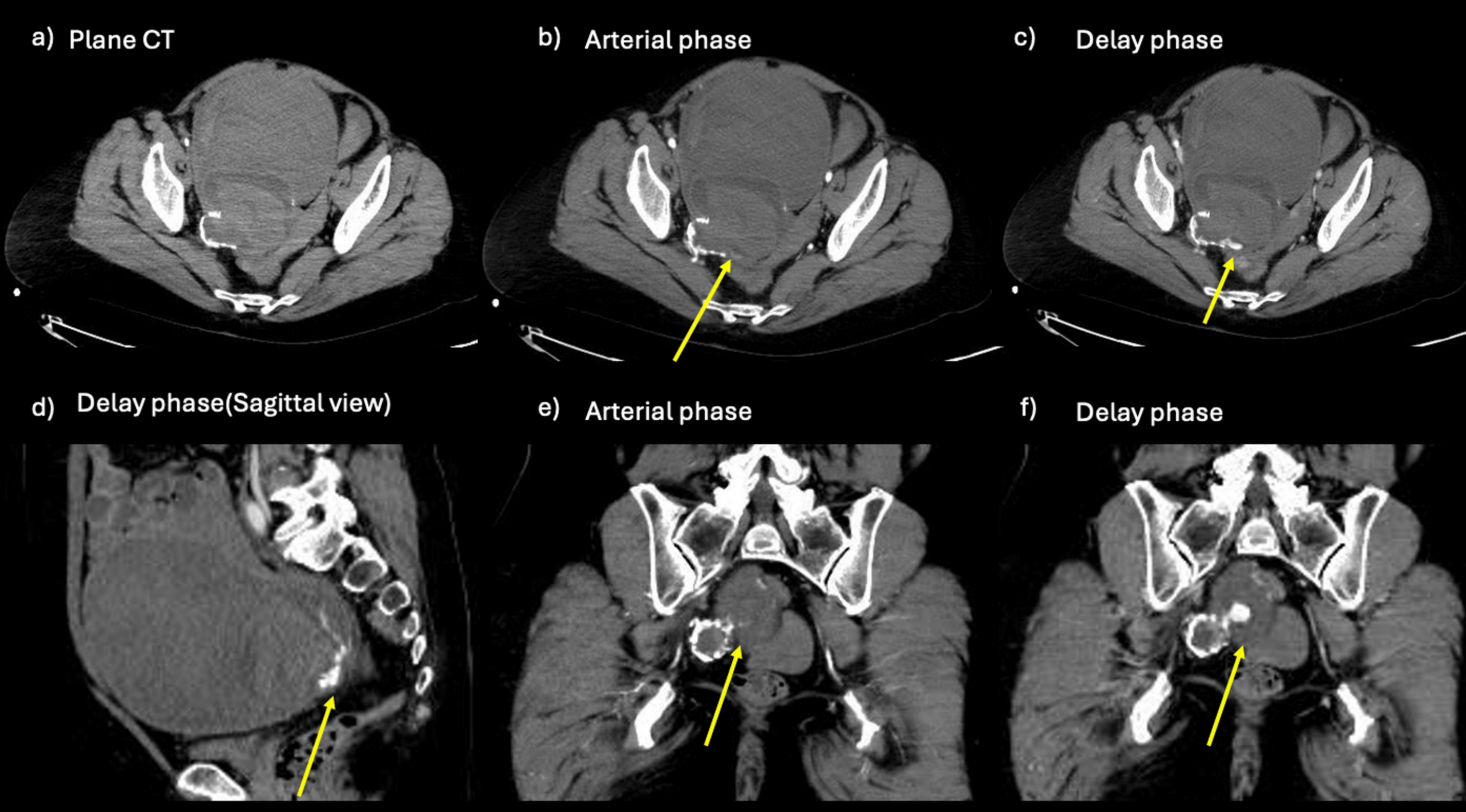
CT images at the previous hospital Although the imaging findings were almost the same as in Figure 2, the extravascular leakage was minute compared to Figure 2. The patient was transferred because the bleeding source was misidentified as ovarian, and hemostasis with transcatheter arterial embolization (TAE) was considered difficult. (a) A well-defined mass lesion with calcification is seen. An even larger structure is visible ventral to the mass lesion (findings unchanged from Figure 2a). (b, e) Contrast-enhanced CT (arterial phase) shows that one part of the lesion lacks calcification. Slight extravascular leakage (arrow) is seen external to the calcified area (unlike Figure 2c, this shows considerably less extravascular leakage). (c, d, f) Contrast-enhanced CT (delayed phase) shows that the extravascular leakage has enlarged (arrow).

The patient was judged to be in hemorrhagic shock, and a massive blood transfusion was immediately started, with six units type O red blood cells (RBCs), six units type AB fresh frozen plasma (FFP), and, after blood type determination, same-type 12 units RBCs and six units FFP were administered. Resuscitative endovascular balloon occlusion of the aorta (REBOA) in Zone III was performed from the right femoral artery, with intermittent occlusion to stabilize the patient’s hemodynamics. Contrast-enhanced CT was performed, and the patient was transferred to our hospital due to intra-abdominal bleeding and suspected bleeding from an ovarian tumor, for which the previous hospital was unable to carry out definitive therapy (Figure 1).

Vital signs on arrival at our hospital were as follows: blood pressure 129/85 mmHg, pulse rate 106 beats per minute, respiratory rate 12 breaths per minute, and SpO₂ 99% (FiO₂ 40%, under ventilator-regulated breathing). The results of blood gas analysis and blood tests at the time of admission showed elevated lactate levels, metabolic acidosis, anemia, and wasting coagulopathy (Table 1).

**Table 1:** Laboratory examination findings PaCO2: partial pressure of arterial carbon dioxide; PaO2: partial pressure of arterial oxygen; HCO3: bicarbonate; BE: base excess; Hb: hemoglobin; Plt: platelet; PT-INR: prothrombin time-international normalized ratio; APTT: activated partial thromboplastin time.

Parameter	Patient value	Reference range
pH	7.032	7.35-7.45
PaCO2 (mmHg）	50.3	35-45
PaO2 (mmHg)	149.9	90-100
HCO3 (mmol/L)	13.3	22-26
BE (mmol/L)	-17.4	-2.5-2.5
Lactate (mmol/L)	10.1	0.56-1.39
Hb (g/dL)	10.5	14-18
Plt (×1000/μL）	5.3	15-40
PT-INR	1.29	0.85-1.15
APTT (seconds)	39.6	20-40
Fibrinogen (mg/dL)	143	180-320
D-dimer (μg/mL)	6.36	＜1

**Figure 2:**
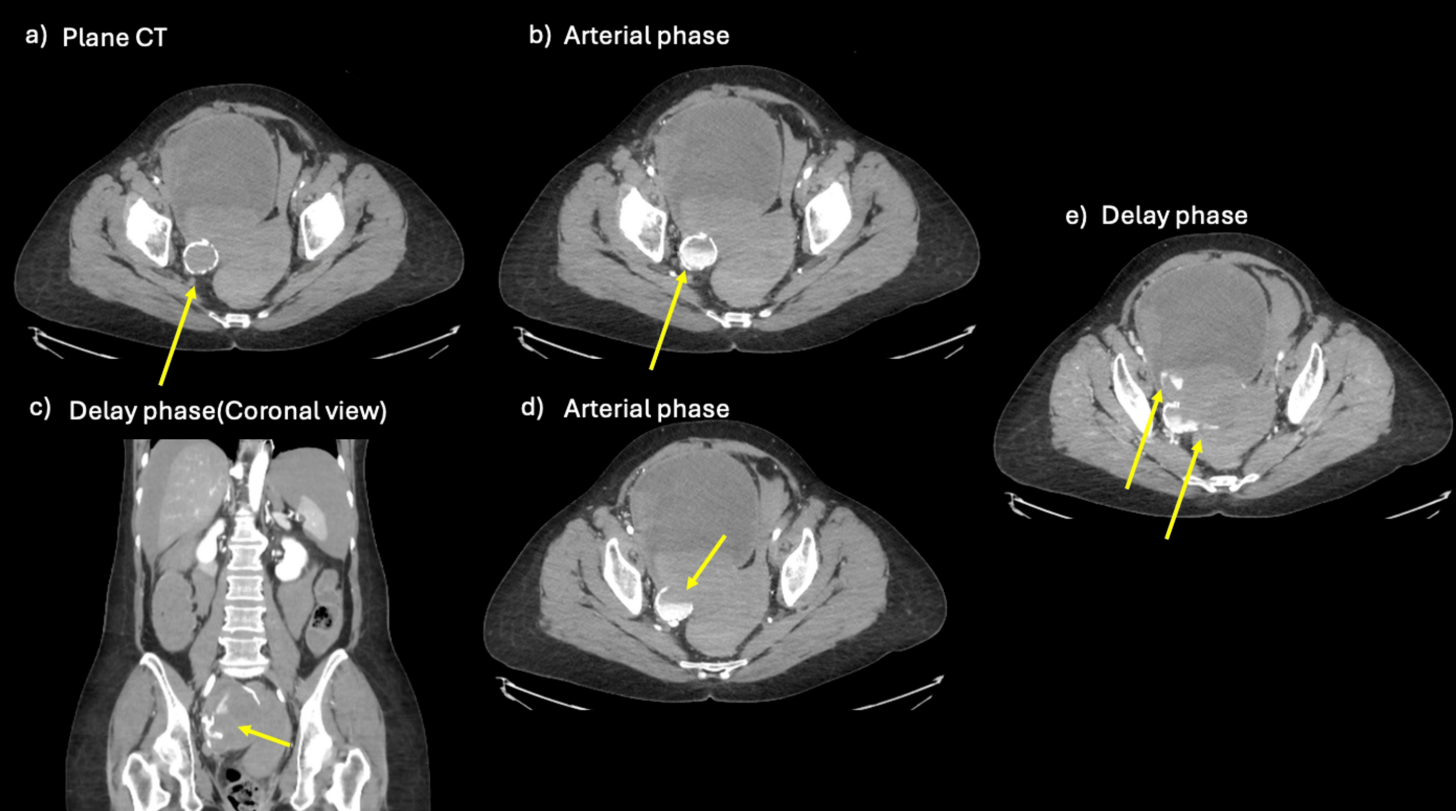
CT images on arrival at our hospital (a) A well-defined mass lesion with calcification is seen (arrow). (b) CT in the arterial phase shows contrast enhancement within a mass lesion containing calcification (arrow). (c) Contrast-enhanced CT (delayed phase) shows that the extravascular leakage has enlarged (arrow). Intra-abdominal hemorrhage is also evident. (d) Contrast-enhanced CT (arterial phase) shows that one part of the lesion lacks calcification, and extravascular leakage is seen external to the calcified area (arrow). This is a finding suggestive of a ruptured pseudoaneurysm. (e) Contrast-enhanced CT (delayed phase) shows that the extravascular leakage has further enlarged (arrow). The extravascular leakage is more evident than in Figure 1, and a diagnosis of rupture of a pseudoaneurysm of the right uterine artery was made.

An additional sheath was secured in the left femoral artery, and contrast-enhanced CT was repeated (Figure 2). 

The CT showed intra-abdominal hemorrhage, a nonenhancing mass in the pelvis, and a massive lesion protruding into the pelvis with a continuous dorsal border of well-defined calcification. The arterial phase showed contrast leakage from part of the calcified lesion; some of the calcified structures were disrupted, and 120 seconds after contrast injection, an enlarging area of extravascular leakage was seen at the same site. TAE was performed based on the assumption that a pseudoaneurysm had formed and ruptured at the right uterine artery transection site.

**Figure 3:**
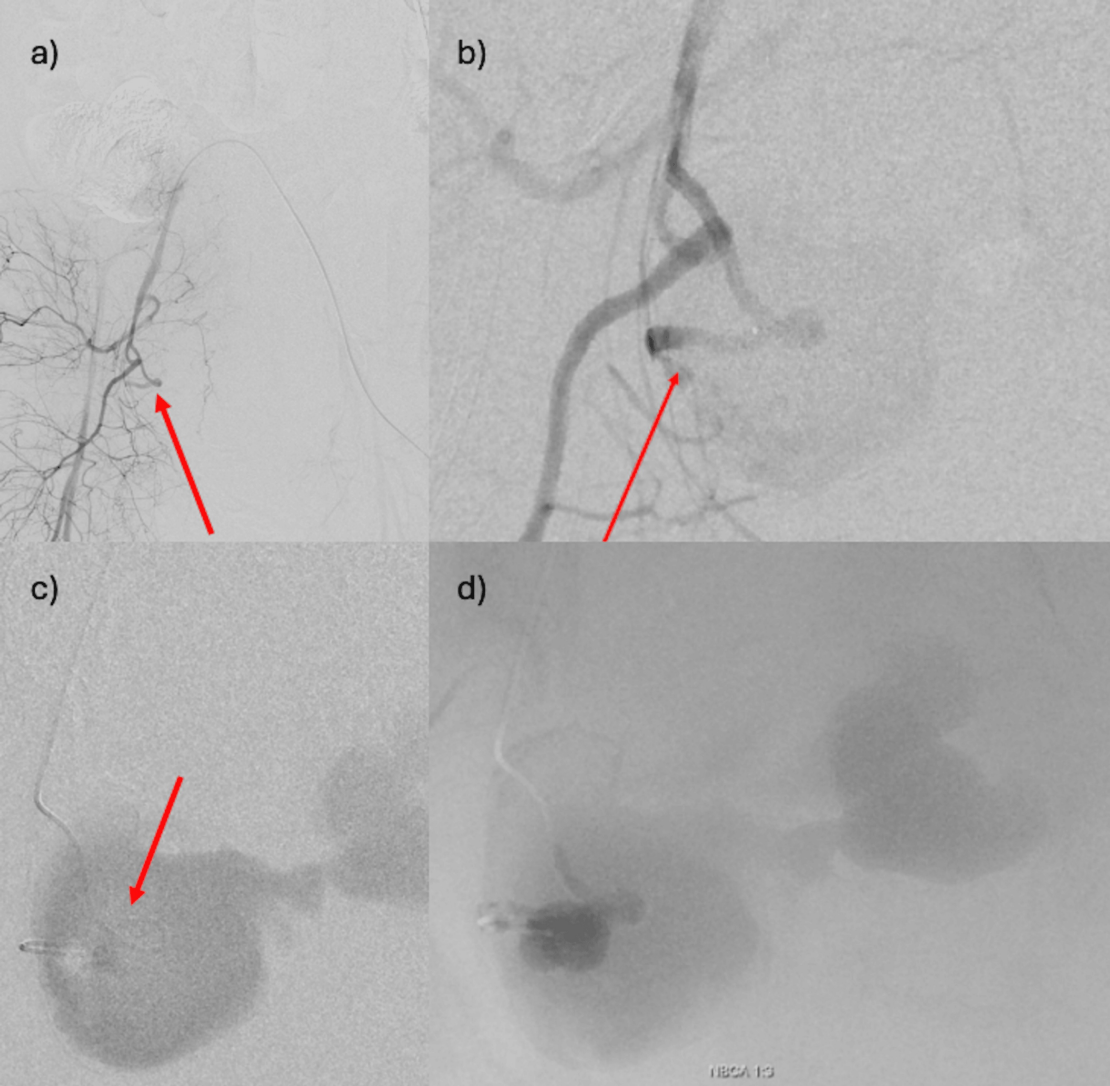
Angiography (a) There is no extravascular leakage, but an abnormal interruption of the uterine artery is observed (arrow). The dilated vessels are abruptly disrupted, suggesting prior damage due to trauma, including surgical manipulation. (b) Selective uterine artery angiography reveals a pseudoaneurysm seeping from the middle of the ascending branch of the uterine artery (arrow). (c) Contrast is seen within the pseudoaneurysm (arrow), and backflow is confirmed, with no flow into the internal iliac artery. (d) Treatment involved embolization with NBCA (N-butyl cyanoacrylate) and Lipiodol in a 1:3 ratio.

Angiography was performed by inserting a 4-Fr Cobra catheter through a sheath in the left femoral artery and selecting the right common iliac artery for angiography. Although no clear extravasation could be identified, the uterine artery appeared to be severed (Figure 3A). After the uterine artery was selected with a microcatheter, uterine arteriography showed a huge pseudoaneurysm that appeared to arise from the middle of the ascending branch (Figure 3B). The contrast images were obtained by advancing the uterine artery catheter to the end of the uterine artery using a double-angle-shaped 0.016-inch, 180-cm ASAHI Meister (ASAHI INTECC, Aichi, Japan), but there was much backflow, and it was difficult to use a liquid permanent embolization material such as NBCA (N-butyl cyanoacrylate).

Therefore, the tip of the microguidewire was reshaped to be more curved, allowing it to pass through the narrow entrance of the pseudoaneurysm and to advance the microcatheter into it. Contrast-enhanced imaging was then performed, highlighting the entire pseudoaneurysm (Figure 3C).

Treatment involved embolization with a 1:3 mixture of NBCA and lipiodol (Figure 3D). 

The patient’s vital signs improved dramatically with TAE. However, there was concern about abdominal compartment syndrome due to significant intra-abdominal hemorrhage. Furthermore, the color tone of the bilateral lower extremities had changed to dark red, and it was determined that there was venous congestion due to hematoma in the pelvis. Therefore, an aspiration kit was placed on the liver surface, and 2.5 liters of intra-abdominal blood were drained. After drainage, the color tone of the lower extremities improved, and abdominal tension also improved.

Since then, the patient has had a good course and left the intensive care unit (ICU) one week after TAE. Pelvic MRI was performed two weeks postoperatively. The reason for MRI was that the wall of the pseudoaneurysm was calcified like a true aneurysm, and the actual mass shown by uniform CT values on the ventral side of the calcification was not clear. It was difficult to differentiate whether it was a residual ovary or a chronically expanding hematoma. There was concern that blood flow into the pseudoaneurysm, including from the ovarian artery, might again resume, and that the mass might re-rupture.

**Figure 4:**
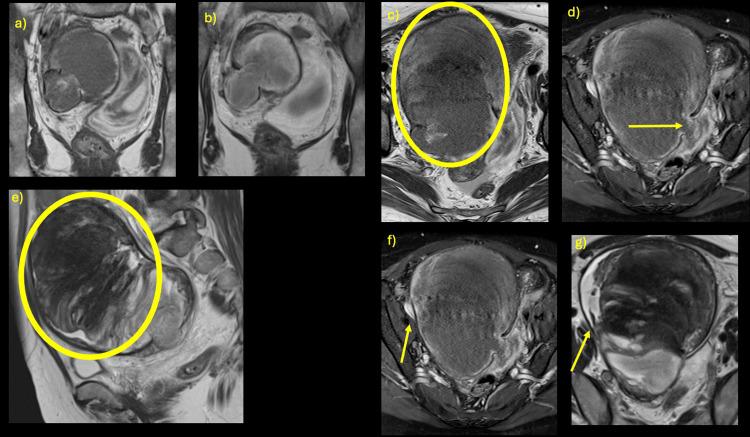
MRI two weeks after TAE (a, b) T1/T2-weighted images (axial view) showing a φ33 mm structure with no signal at the outer edge: first pseudoaneurysm (outer edge calcified). (c) T1-weighted image (axial view) showing a partially signal-free area that is torn off, and the structure has diffusion limitation equivalent to that within the mass from ventral to left (circle). (d) T1-weighted image with fat suppression (axial view) showing a high signal intensity outer edge: subacute hematoma (site of this rupture). (e) T2-weighted image (sagittal view) showing the ventral side of the aneurysm with a layered heterogeneous signal as if folded, with no enhancement, suggestive of an organized intra-aneurysmal thrombus (circle). (f, g) T1-weighted image with fat suppression and T2-weighted image (axial view) showing high signal intensity of the right margin of the mass, which suggests a residual, nonorganized area (arrow).

Magnetic resonance imaging (MRI) showed a 3 cm diameter structure on the right side of the pelvic cavity with no signal at the outer edge in either sequence, suggesting a pseudoaneurysm and calcification of the outer edge. The outer edge was partially transected, and there was a series of structures with diffusion restriction equivalent to that within the mass, extending from the ventral side to the left side. The ventral mass was determined to be a pseudoaneurysm after a contained rupture, over 16 cm in diameter. The ventral side within the aneurysm showed a layered heterogeneous signal and was considered to be an organic intra-aneurysmal thrombus with no enhancement. On the right side, however, T2-weighted and fat-suppressed T1-weighted images showed high signal intensity, suggesting residual nonorganized areas (Figure 4).

Therefore, surgical resection and repair were recommended because of the residual, nonorganized areas and massive mass wall rupture. However, when radical surgery was requested from the gynecology department, they recommended conservative treatment because of the high risk.

The patient herself wanted to undergo radical surgery, knowing the risks, if there was a possibility of re-rupture and life-threatening bleeding. Therefore, she consulted the hospital where she had undergone her first surgery 20 years earlier for a second opinion and underwent a laparotomy to remove a pelvic mass six months after the rupture.

The initial intraoperative appearance was a huge pseudoaneurysm arising from the right side of the vaginal disc. Internally, there was only an old hematoma and no active bleeding. Although the hematoma could be removed, the wall of the pseudoaneurysm was adherent to the right pelvic wall, and because of the risk of ureteral injury, the gynecologist did not remove the aneurysm completely but resected it partially, leaving part of the pseudoaneurysm wall in the pelvis, and the operation was completed. The patient did not experience further bleeding after the surgery and was discharged from the hospital on the seventh day. The remaining pseudoaneurysm in the pelvis was closed by suturing the remaining aneurysm wall, and the maximum diameter of the aneurysm was about 4.5 cm. The patient remains under outpatient follow-up with the expectation of natural resorption.

## Discussion

Pseudoaneurysms of the uterine artery may be nontraumatic, caused by pregnancy itself. Baba et al. reported from 3 to 6 cases per 1000 deliveries [6]. Although this frequency is surprisingly high, they noted that they rarely rupture and may resolve spontaneously [7]. Most UAPs are traumatic, following repeat curettage, abortion, cesarean section, or gynecological surgery, with rupture being reported to be rare [2,3,5]. The approximate time from pelvic surgery to symptom onset is usually one week to three months. This delay is said to be due to the gradual increase in the size of the pseudoaneurysm caused by the characteristic increase in pressure. Blood flow to the pseudoaneurysm is more systolic than diastolic, resulting in a gradual increase in pressure and eventual rupture [8]. For the diagnosis of unruptured UAPs, transvaginal ultrasonography is commonly used. The pseudoaneurysm presents with a characteristic sonographic image consisting of a pulsating, anechoic or hypoechoic, well-defined, cystic structure. Color Doppler ultrasonography can help confirm the diagnosis by showing blood flow within the cystic structure after identification on grayscale, but sensitivity varies by site. Angiography is widely used for definitive diagnosis and treatment, as it can lead directly to endovascular intervention [9].

Rupture of a UAP is potentially fatal, and treatment by endovascular embolization is currently the preferred management for cases that do not heal spontaneously [3]. Size is said to be of little relevance with regard to rupture [10]. Baba et al. found that UAP rupture may depend on the balance between blood flow or pressure within the UAP and the strength of the UAP wall. They proposed that "lack of diastolic flow" may predict spontaneous UAP resolution [7]. In the present case, the UAP was not diagnosed prior to rupture, and early detection remained a challenge. The technical success rate of endovascular treatment has been shown to be as high as 97% [9]. Because failure to detect bleeding by angiography does not significantly affect the success rate of uterine artery embolization, uterine artery embolization is recommended even when bleeding is not confirmed by angiography [11,12]. The present case was identified 20 years after surgery for uterine fibroids, presenting with symptoms of abdominal pain and shock.

Compared with previous reports, the time to manifest symptoms due to UAP in this case is characterized by a very long interval [13] (Table 2). 

**Table 2:** Comparison with previous cases of uterine artery pseudoaneurysm (UAP) rupture

Study	Surgery	Surgical indication	Age at surgery, years	Signs and symptoms caused by uterine artery pseudoaneurysm	Interval between surgery and symptom onset	Diagnostic procedures	Initial diagnosis	State of pseudoaneurysm at diagnosis	Size, mm	Location of pseudoaneurysm	Treatment procedure
Langer and Cope［2］	Total vaginal hysterectomy	Uterine prolapse and urinary retention	40	Left lower quadrant abdominal pain, fever, chills, and nausea	6 days	CT, pelvic sonography, color Doppler sonography, pelvic arteriography	Pelvic abscess	Unruptured	20	The remaining portion of the left uterine artery	Transarterial embolization
Lee et al［3］	Total abdominal hysterectomy and bilateral salpingo-oophorectomy	Cervical intraepithelial neoplasia, grade III, and adenocarcinoma in situ of a cervical polyp	49	Fever, lower abdominal pain initially, then hemorrhagic shock	4 days, 16 days	Pelvic sonography, color Doppler sonography, pelvic arteriography	Urinary tract infection, then pseudoaneurysm	Ruptured	25	Uterine artery stump	Transarterial embolization
Higón et al［5］	Abdominal myomectomy	Intramural myoma	40	Sudden and abundant metrorrhagia	40 days	Pelvic sonography, color Doppler sonography, pelvic arteriography	Ruptured pseudoaneurysm	Ruptured	30	Left uterine artery	Transarterial embolization
Takeda et al［13］	Laparoscopic-assisted myomectomy	Intramural myoma	32	Sudden massive uterine hemorrhage at menstruation	79 days	Pelvic sonography, pelvic arteriography	Massive uterine hemorrhage of unknown origin	Ruptured	29	The peripheral branch of the left uterine artery	Transarterial embolization
Current report	Abdominal hysterectomy	Intramural/intramuscular myoma	32	Sudden abdominal pain initially, then hemorrhagic shock	20 years	CT, pelvic arteriography	Ruptured ovarian tumor suspected	Ruptured	30,160	Uterine artery stump	Transarterial embolization/open hematoma removal, partial excision of pseudoaneurysm

This point is more important than previous reports because a longer follow-up is needed, or patients need to be educated about it. However, the retroperitoneal and intravaginal hematoma observed one week after the initial surgery may have been an early symptom of UAP, which is consistent with the timing of symptom appearance in previous reports. A 3 cm cyst in the right pelvis on CT at one year postoperatively could have been considered a cystic component if not for the contrast-enhanced CT.

The site of the UAP in the present case is thought to have originated in the vaginal stump and uterine isthmus, based on the first surgical record. This is consistent with the fact that the myometrium is often treated conservatively, and the probability of rupture is about 57%, typically presenting as massive hemorrhage after trauma such as surgery. However, when the organic portion is included, the size of the UAP in the present case was as large as 16 cm in diameter, and such a large pseudoaneurysm has never been reported in the past. It was not a single pseudoaneurysm but a pseudoaneurysm of about 3 cm in diameter with calcification that repeatedly underwent oozing rupture and formed a huge mass in the pelvic cavity. The reason why the mass increased in size without rupturing for such a long period remains unknown. Further evaluation of similar cases is needed.

Direct treatment for the rupture was endovascular therapy. Technically, the uterine artery had a pseudoaneurysm at the ruptured end, and its entrance was quite narrow. This made it difficult to use a liquid embolization material such as NBCA, which could leak out of the uterine artery and result in unintended wide embolization. However, there were concerns that embolization of the uterine artery origin using metal coils would be difficult if the pseudoaneurysm had other incoming vessels, so there was a desire to refrain from using this technique. Since the catheter could be advanced into the pseudoaneurysm, it was possible to contrast only within the calcified pseudoaneurysm without leakage of contrast medium in front of it, and thus NBCA could be used to embolize it. Furthermore, it is worth reporting that the combination of surgery and NBCA was able to reduce the number of residual pseudoaneurysms that might rupture again in the future to the maximum extent possible. Hybrid treatment with surgery may be useful depending on the size, location, and cause of the pseudoaneurysm, in addition to endovascular treatment.

## Conclusions

A late complication after myomectomy decades later is rupture of a UAP. Although treatment with TAE is highly effective, it is advisable to consider embolization methods that avoid proximal embolization in the case of large pseudoaneurysms. Additional surgery is an option for large pseudoaneurysms that occupy the pelvis, even if rebleeding is not observed after embolization. Thus, emergency physicians and gynecologists need to recognize that a combination of interventional radiology (IVR) and surgery is crucial for radical treatment.
